# *Listeria monocytogenes* in Fruits and Vegetables: Antimicrobial Resistance, Biofilm, and Genomic Insights

**DOI:** 10.3390/antibiotics13111039

**Published:** 2024-11-03

**Authors:** María Guadalupe Avila-Novoa, Oscar Alberto Solis-Velazquez, Pedro Javier Guerrero-Medina, Liliana Martínez-Chávez, Nanci Edid Martínez-Gonzáles, Melesio Gutiérrez-Lomelí

**Affiliations:** 1 Centro de Investigación en Biotecnología Microbiana y Alimentaria, Departamento de Ciencias Básicas, División de Desarrollo Biotecnológico, Centro Universitario de la Ciénega, Universidad de Guadalajara, Av. Universidad 1115, Col. Lindavista, Ocotlán 47820, Jalisco, Mexico; avila.novoa@cuci.udg.mx (M.G.A.-N.); oscar_sv_086@hotmail.com (O.A.S.-V.); pjgm@cuci.udg.mx (P.J.G.-M.); 2 Departamentos de Farmacobiología y Matemáticas, Centro Universitario de Ciencias Exactas e Ingenierías, Universidad de Guadalajara, Blvd. Gral. Marcelino García Barragán 1451, Col. Olímpica, Guadalajara 44430, Jalisco, Mexico; liliana.mchavez@academicos.udg.mx (L.M.-C.); nanci.martinez@academicos.udg.mx (N.E.M.-G.)

**Keywords:** *Listeria monocytogenes*, virulence factors, antimicrobial resistance, benzalkonium chloride, biofilms

## Abstract

Background/Objectives: *Listeria monocytogenes* is a foodborne pathogen that can infect both humans and animals and cause noninvasive gastrointestinal listeriosis or invasive listeriosis. The objectives of this study were to determine the genetic diversity of *L. monocytogenes*; the genes associated with its resistance to antibiotics, benzalkonium chloride (BC), and cadmium chloride (CdCl_2_); and its biofilm formation. Methods: A total of 132 fresh fruits (44 samples) and vegetables (88 samples) were selected for this study. The genetic diversity of the isolates and the genes associated with their antibiotic resistance were determined using PCR amplification; meanwhile, their levels of susceptibility to antibiotics were determined using the agar diffusion method. Their levels of resistance to BC and CdCl_2_ were determined using the minimum inhibitory concentration method, and their capacity for biofilm formation was evaluated using the crystal violet staining method. Results: A total of 17 *L. monocytogenes* strains were collected: 12.8% (17/132) from fresh fruits and vegetables in this study. The isolates of *L. monocytogenes* belonged to phylogenetic groups I.1 (29.4% (5/17); serotype 1/2a) and II.2 (70.5% (12/17); serotype 1/2b); strains containing *Listeria* pathogenicity islands (LIPIs) were also identified at prevalence rates of 100% for LIPI-1 and LIPI-2 (17/17), 29.4% for LIPI-3 (5/17), and 11.7% for LIPI-4 (2/17). The antibiotic susceptibility tests showed that the *L. monocytogenes* isolates exhibited six different multiresistant patterns, with multiple antibiotic resistance (MAR) index of ≥0.46 (70.5%; 12/17); additionally, the genes *Ide, tetM,* and *msrA*, associated with efflux pump Lde, tetracycline, and ciprofloxacin resistance, were detected at 52.9% (9/17), 29.4% (5/17), and 17.6% (3/17), respectively. The phenotypic tests showed that 58.8% (10/17) of cadmium-resistant *L. monocytogenes* isolates had a co-resistance of 23.5% (4/17) to BC. Finally, all strains of *L. monocytogenes* exhibited moderate biofilm production. Conclusions: The results of this study contribute to our understanding of the persistence and genetic diversity of *L. monocytogenes* strains isolated from fresh fruits and vegetables; in addition, their resistance to CdCl_2_, which is correlated with co-resistance to BC disinfectant, is helpful for the food industry.

## 1. Introduction

*Listeria monocytogenes* is a facultative intracellular pathogen, widely distributed in nature, that causes noninvasive gastrointestinal listeriosis or invasive listeriosis. The clinical manifestations of invasive listeriosis include septicemia, encephalitis, endocarditis, meningitis, abortions, and fetal death, while noninvasive gastrointestinal listeriosis may be asymptomatic or include only flu-like symptoms or febrile gastroenteritis syndrome [1,2]. Invasive listeriosis affects high-risk groups, including populations such as elderly adults (>65 years), pregnant people, newborns, immunocompromised people, and patients with cancer or diabetes [3,4]. The Centers for Disease Control and Prevention (CDC) estimates that there are approximately 1600 cases of listeriosis, causing 260 deaths, annually [3]. In the European Union (EU), there are approximately 2500 cases of invasive listeriosis in humans annually, and it is the most serious cause of foodborne disease, with high rates of hospitalization and death; additionally, the zoonosis of this pathogen in 2022 increased by 15.9% compared to 2021, with 2738 cases [4].

*L. monocytogenes* can be spread through the agricultural environment, such as soil and irrigation water, as these sources may contaminate fresh produce, including fruits and vegetables at various stages of production and processing [5,6,7], thereby creating a public health problem and economic losses for the food industry.

In fact, the Interagency Food Safety Analytics Collaboration (IFSAC) reports that 76% of foodborne *L. monocytogenes* illnesses (2016–2020) in the United States were associated with three categories in particular: dairy products (37.1%), fruits (24.8%), and row vegetable crops (14.1%) [8]. The United States Food and Drug Administration Recalls, Market Withdrawals, & Safety Alerts publication reported recalls of fruits and vegetables in 2023 due to the presence of this pathogen in organic green kiwifruit, organic frozen pineapple, and frozen fruit blends containing organic frozen pineapple, kale, spinach, collard green products, and mung bean sprouts [9]. Furthermore, Food Standards Australia–New Zealand (FSANZ), during the period from 2019 to 2023, reported 83 food recalls associated with contamination by foodborne pathogens, of which *L. monocytogenes* represented 36% (30 recalls), followed by *Salmonella* spp. (33%; 27 recalls) and *Escherichia coli* (22%; 18 recalls); the recalls included several food categories, such as fruits and vegetables, dairy products, meats and processed meats, etc. [10].

Additionally, the prevalence of resistant *L. monocytogenes* isolates in food and the environment has been associated with the use of antibiotics in medicine, veterinary medicine, and agricultural production systems, as well as with practices such as treating soils with manure or growth promoters and the misuse of therapeutic treatments for veterinary purposes [11,12,13]. The increase in multiresistant pathogens is a significant public health problem, a situation that becomes increasingly severe as these pathogens spread globally and acquire new resistance mechanisms until there are no alternative therapies for their control [14,15]. In fact, the World Health Organization estimates that bacterial resistance will cause 10 million deaths by 2025 [16]. Moreover, the persistence of *L. monocytogenes* is linked to the following further issues: (i) resistance to antimicrobials or sanitizing treatments; (ii) the ability of cells to form biofilm on equipment or in the surrounding environment; (iii) the survival of strains under various food preservation conditions or environmental stresses; and (iv) the inability to remove cells from niches in the food environment [17,18,19,20]. Therefore, the objectives of the present study were to determine the following: (i) the genetic diversity of *L. monocytogenes* isolated from fresh fruits and vegetables; (ii) the genes associated with antibiotic- and multidrug-resistant strains of *L. monocytogenes*; and (iii) the resistance of some strains to benzalkonium chloride (BC) and cadmium chloride (CdCl_2_) and their biofilm formation capacity.

## 2. Results

### 2.1. Genomic Profile of L. monocytogenes Sublineages and Virulence Genes

In total, 17 *L. monocytogenes* isolates were obtained from the 17 positive samples (12.8%: 1.5% cilantro, 2.2% broccoli, 3.7% lettuce, and 5.3% Hass avocados), while 115 (86.9%) out of 132 samples were negative (qualitative detection (limit of detection < 1 CFU per analytical unit)) for *L. monocytogenes*. The isolates of *L. monocytogenes* belonged to phylogenetic groups I.1 (29.4% (5/17); serotype 1/2a) and II.2 (70.5% (12/17); serotype 1/2b). Additionally, pathogenicity islands were detected in *L. monocytogenes*, including LIPI-1 and LIPI-2 (100%; 17/17), LIPI-3 (29.4%; 5/17), and LIPI-4 (11.7%; 2/17); among the isolates, the following pathogenicity islands were found: LIPI-1 + LIPI-2 (70.5% (12/17)), LIPI-1 + LIPI-2 + LIPI-3 (17.6% (3/17)), and LIPI-1 + LIPI-2 + LIPI-3 + LIPI-4 (11.7% (2/17)) (Table 1). The *prfA* and *actA* genes were detected in 100% (17/17) of the isolates.

### 2.2. Antimicrobial Resistance Gene Profiling

A total of 17 isolates were screened for the ciprofloxacin resistance gene *Ide* (52.9%; 9/17), of which 41.1% (7/17) showed intermediate phenotypic resistance, and 11.7% (2/17) showed resistance to ciprofloxacin. The tetracycline resistance gene *tetM* was detected in 29.4% (5/17), whereas five isolates showed phenotypic resistance, and the macrolide resistance gene *msrA* was detected in 17.6% (3/17). However, neither the macrolide resistance gene *ermA* nor the chloramphenicol resistance gene *cat* was detected (Table 1).

### 2.3. Antimicrobials, Sanitizing, Cadmium, and Biofilm

*L. monocytogenes* was found to be susceptible to ciprofloxacin (47%; 8/17), tetracycline (70.5%; 12/17), gentamicin (94.1%; 16/17), erythromycin (94.1%; 16/17), vancomycin (100%; 17/17), chloramphenicol (100%; 17/17), and trimethoprim–sulfamethoxazole (100%; 17/17), according to Table 2; however, the isolates were resistant to first- and third-generation β-lactams (penicillin, ampicillin, dicloxacillin, cephalothin, and cefotaxime) (94.1–100%; 16–17/17), clindamycin (52.9%; 9/17), tetracycline (29.4%; 5/17), and ciprofloxacin (11.7%; 2/17). Among the tested *L. monocytogenes* isolates, six different multiresistant patterns were observed, with the most common being PE-CF-AM-CFX-DC (23.5%; 4/17) and PE-CF-AM-CFX-DC-CLM (35.2%; 6/17). The MAR index for *L. monocytogenes* isolates ranged from 0.23 to 0.61, with 70.5% (12/17) presenting a MAR index of ≥0.46 (Table 1). Additionally, intermediate clindamycin and ciprofloxacin resistance was present in 41.1% (7/17) (Table 2). In addition, the MICs of 76.4% (13/17) of the isolates was between 0.7 and 3.1 µg/mL BC, while the criterion for resistance to BC is CMI ≥ 6 µg/mL, at least twice the MIC for the predominant number of *L. monocytogenes* strains (MIC = 3.1 µg/mL) (Table 3). The MICs of 41.1% (7/17) of the isolates were <70 µg/mL CdCl_2_, and 58.8% (10/17) were cadmium-resistant *L. monocytogenes* strains (MIC ≥ 70 µg/mL). Finally, all *L. monocytogenes* were classified as moderate biofilm producers (Table 3).

## 3. Discussion

*Listeria monocytogenes* is a foodborne pathogen that causes invasive or noninvasive listeriosis in humans; the severity of the pathogenesis is associated with several factors, including risk groups and hazard characterization. In the present study, 12.8% of fresh fruits and vegetables tested were contaminated with *L. monocytogenes* strains belonging to serotypes 1/2a (29.4%) and 1/2b (70.5%). Several authors [5,7,21,22] have reported similar prevalence percentages for serotypes 1/2a–3a (33–65%) and 1/2b-3b-7 (50–79.6%); however, Maćkiw et al. [22] and Chen et al. [23] identified a lower prevalence for serotypes 1/2a–3a (10.8%) and 1/2b (2%) among the isolated *L. monocytogenes* found in ready-to-eat (RTE) foods, fruits, and fresh and frozen vegetables. Likewise, Kayode and Okoh [7] did not detect serotype 1/2a, but 1/2b (79.61%) and 4b (8.7%) were detected among *L. monocytogenes* strains found in fruit and vegetable samples. Indeed, serotypes 1/2a, 1/2b, 1/2c, and 4b are responsible for 95% of human listeriosis cases and have been frequently isolated from food products and patients [5,24,25], suggesting that the diversity of *L. monocytogenes* serotype prevalence may be related to geographic regions, monitoring procedures, methodologies for detecting of *L. monocytogenes* in various food categories (fruits, vegetable row crops, dairy, pork, chicken, beef, etc.), specific characteristics of the food (fresh or frozen), or sources of contamination that interact during food production and distribution.

In this investigation, we detected the presence of *L. monocytogenes* pathogenicity islands (LIPIs) in order to assess the potential risks that *L. monocytogenes* may pose to public health. All strains of *L. monocytogenes* isolated from fresh fruits and vegetables were found to have *Listeria* pathogenicity island 1 (LIPI-1; *prfA, hly, plcA, plcB, mpl,* and *actA*), which encodes virulence factors that promote the growth and spread of *L. monocytogenes*. Once inside the host cell, the phagocytic vacuole is lysed by listeriolysin O (LLO), which is a pore-forming toxin encoded by the *hly* gene that mediates the lysis of bacterial cells in the host cytoplasm and enhances its cytolytic action through phosphatidylinositol-PLC and phosphatidylcholine-PLC, which mediate pathogen escape from single- and double-membrane-bound vacuoles. ActA plays a role in facilitating the motility of the bacterial cell to the host cell’s cytoplasm, and the actin cytoskeleton is hijacked to favor cell-to-cell spread [26,27,28]. The *prfA* gene, which encodes the PrfA regulatory protein that controls the expression of the pathogenicity determinants of *L. monocytogenes*, was also identified in this study [29]. Our results are similar to those reported by several other researchers [5,7,30,31], showing that *prfA* (100%), *mpl* (92–100%), *plcA* (92–100%), *plcB* (100%), *hly* (100%), and *actA* (84–100%) were detected in the *L. monocytogenes* strains isolated from fruits and fresh and frozen vegetables, as well as agricultural environments such as irrigation water and agricultural soil. *Listeria* pathogenicity island 2 (LIPI-2; *inlA*, *inlB*, *inlC*, and *inlJ*) was detected in all the *L. monocytogenes* strains identified in this study, which is in agreement with several prior investigations detecting the genes *inlA* (74.1–100%), *inlB* (81.5–100%), *inlC* (70.6–100%), and *inlJ* (66.7–100%), which encode a set of internalins that play roles in the adhesion and invasion of *L. monocytogenes* cells to the host cells, as well as *L. monocytogenes* dissemination [5,7,22,29,30,31]. InlA adheres to and invades intestinal epithelial cells that express the E-cadherin receptor, thereby facilitating intestinal barrier crossing; additionally, InlC and InlJ are involved in the postintestinal dissemination of *L. monocytogenes* infection [6,22,32].

Other islands detected in this study were *Listeria* pathogenicity island 3 (LIPI-3), in 29.4%, and *Listeria* pathogenicity island 4 (LIPI-4), in 11.7% of the samples. LIPI-3 encodes listeriolysin S (LLS), a bacteriocin with hemolytic and cytotoxic factors that contributes to polymorphonuclear neutrophil survival as well as to alteration in the gut microbiota [22,30,33], while LIPI-4 is involved in the infection of the host’s neuronal and placental tissues, in addition to conferring potential hypervirulent strains [34,35].

Additionally, all *L. monocytogenes* isolates detected in this study exhibited antibiotic resistance, with penicillin, ampicillin, dicloxacillin, cephalothin, cefotaxime, clindamycin, tetracycline, and ciprofloxacin resistance being the most frequently encountered. Several studies have reported varying prevalences of antimicrobial resistance to penicillin (2.5–100%), ampicillin (50–100%), gentamicin (20–40%), STX (30%), erythromycin (23.5–100%), tetracycline (90–100%), chloramphenicol (20–70%), cefotaxime (80–100%), clindamycin (57.5–100%), cephalothin (50–100%), and ciprofloxacin (35.2–40%) in *Listeria* spp., and particularly in *L. monocytogenes* isolates from different categories of food and food processing environments [31,36,37,38]. The intermediate phenotypic resistance to clindamycin (41%), ciprofloxacin (41%), erythromycin (5.8%), and gentamicin (5.8%) demonstrated in the *L. monocytogenes* isolates in this study is also in agreement with the findings of other investigators [37,38,39], who reported the prevalences of intermediate resistance to clindamycin (30%), ciprofloxacin (5–64.7%), gentamicin (2.5%), and tetracycline (2–5.8%) in *L. monocytogenes.* The overprescription of antibiotics in clinical practice, the use of antibiotics in animal production, inadequate veterinary treatment for preventing animal disease, and the migration and accumulation of veterinary antibiotic residues in agricultural soils and irrigation water may contribute to the antimicrobial resistance and heterogeneity in the prevalence levels of the patterns observed in *L. monocytogenes* isolates [11,13,38,40].

The results of this study demonstrate that *L. monocytogenes* strains exhibit six different multiresistant patterns, with a MAR index of ≥0.46 (70.5%), indicating a higher risk of the source having been exposed to antibiotics, as a MAR index of ≥0.2 suggests the intensive use of antibiotics in the region and a high risk of promoting antibiotic resistance [41,42]. Iwu and Okoh [31], as well as Maurice Bilung et al. [36], reported similar MAR index values (0.31–0.85) for multidrug-resistant *Listeria* spp. and for *L. monocytogenes* isolates from irrigation water and agricultural soil (0.2–1), suggesting a high-risk source that is constantly exposed to antibiotics that are used to prevent or treat animal disease and promote animal growth. Agricultural activities such as the use of fertilizers containing antibiotic residues increase antibiotic resistance as well as the prevalence of antibiotic-resistant strains in the soil and in the water used for plant irrigation [12,43].

Furthermore, *L. monocytogenes* has been found to develop resistance to several antibiotics, including tetracycline, ciprofloxacin, erythromycin, clindamycin, penicillin, and ampicillin, through the acquisition of genetic elements such as conjugative transposons and self-transferable or mobilizable plasmids [13,28,44]; therefore, the detection of *Ide*, *tetM*, and *msrA* in the *L. monocytogenes* isolates in our study may be related to resistance mechanisms such as efflux pump Lde and transposon Tn916 harboring *tetM*, which confer resistance to ciprofloxacin and tetracycline [44,45], which is in accordance with the detection of *Ide, tetM*, and *msrA* in *Listeria* spp. and *L. monocytogenes* isolates from slaughtering and processing environments, both food-related and clinical [7,15,39,46]. Although we did not detect the presence of *cat* or *erm*A genes among our *L. monocytogenes* isolates, *cat* (100%) and *ermA* (16.9%) have already been detected among *Listeria* spp. in food and processing environments [15,46]. In fact, these mechanisms of resistance affect the treatment of human listeriosis with regard to drugs such as (*i*) first-line ampicillin or penicillin G in combination with an aminoglycoside (gentamicin) and (*ii*) second-line trimethoprim in combination with a sulfonamide, such as sulfamethoxazole-co-trimoxazole, as well as erythromycin, tetracycline, and vancomycin [28,45].

This investigation suggests that the high prevalence of resistance and intermediate resistance in *L. monocytogenes* isolates may be due to the inadequate use of antimicrobial agents in veterinary medicine, the extensive use of animal foodstuffs, agricultural production systems, or the intrinsic resistance of *L. monocytogenes* to cephalosporins and fluoroquinolones, which is associated with the lack or low affinity of the enzyme that catalyzes the final step of cell wall synthesis [28,45]. However, the prevalence of antibiotic resistance reported in different countries is influenced by the health policies related to comprehensive antimicrobial management and the determination of antimicrobial breakpoints specific to veterinary medicine, particularly regarding the methods for antimicrobial susceptibility testing for bacterial pathogens of animal origin and zoonotic bacteria that can affect humans [39,47].

Moreover, *L. monocytogenes* has demonstrated resistance to nonessential toxic metals, including arsenic and cadmium [48]. In this study, 58.8% of the *L. monocytogenes* isolates were resistant to CdCl_2_ (MIC ≥ 70 µg/mL); this was a particularly common occurrence among the isolates of serotypes 1/2a and 1/2b, and similar findings have been reported by other researchers [30,49,50,51] regarding the prevalence of cadmium resistance (63–90%) in serotypes 1/2a and 1/2b of *L. monocytogenes* isolated from food and the environment. The presence of heavy metal residues in the environment is related to anthropogenic sources, including the industrial sector, as well as agricultural practices such as using phosphate fertilizers, which represent significant sources of cadmium in agricultural soil, water, and food [52], thus increasing the survival potential of *L. monocytogenes* by inducing the acquisition of mobile genetic elements of heavy metal resistance determinants in diverse environmental niches. Likewise, Zhang et al. [51] argued that cadmium exerts long-term selective pressure, allowing *L. monocytogenes* to develop tolerance.

On the other hand, QACs are used in the food industry during disinfection processes to control, reduce, and inactivate foodborne pathogens [53,54,55,56]; however, the prevalence of QAC resistance, particularly to BC, has been detected in *L. monocytogenes* isolated from food and processing plant environments and is associated with cadmium [57,58,59,60]. Our research showed that four *L. monocytogenes* isolates (23.5%) were resistant to BC, and ten isolates (58.8%) were resistant to cadmium with co-resistance to BC and cadmium (23.5%). Ratani et al. [61] isolated strains that showed 14% resistant to BC and 57% to Cd, while Xu et al. [50] detected 16.7% resistant to BC and Cd in *L. monocytogenes*; however, the cadmium- and BC-resistant *L. monocytogenes* were not always correlated [49]. Based on the cadmium or BC resistance results, this result could be due to the genetic diversity of the *L. monocytogenes* strains associated with the genetic determinants of cadmium resistance, such as *cadA*1 (plasmid-transposon Tn5422), *cadA*2 (plasmid pLM80), *cadA3* (at the chromosome level of *L. monocytogenes*), and *cadC* [48,62]; or BC resistance, such as qac*A/B*, *qacC/D*, *qacE*, *qacE1Δ-sul*, *qacF*, *qacG*, *bcrABC*, transposon Tn6188 (containing the *qacH* gene), or *mdrL* (chromosome- and plasmid-borne; encodes an efflux pump) [50,60,63,64]. In addition to the various breakpoints specific to determining resistance to disinfectants (MIC = 4–32 µg/mL), this multitude of factors may interfere with the prevalence of resistance phenomena regarding BC in *L. monocytogenes*, as they are established according to the number of *L. monocytogenes* isolates, the origins of strains, the susceptibility testing medium, etc. [65].

Additionally, the decrease in QAC efficiency is related to (*i*) environmental niches, with sites that are difficult to clean and disinfect, along with the inability to remove cells; (*ii*) the presence of organic matter on food contact surfaces; (*iii*) exposure to sublethal concentrations of QACs on food contact surfaces that, in turn, confer BC tolerance to *L. monocytogenes*. Moreover, this phenomenon is associated with the persistence of *L. monocytogenes* in the food industry, along with the subsequent adaptation and formation of biofilms [18,66,67,68]. Our results indicate that all *L. monocytogenes* isolates can form biofilms, as they harbor the genes *inlA*, *prfA, plcA*, *hly*, *plcB*, and *actA*, which are associated with biofilm formation. Previous research indicated that *inlA*, *inlL*, *prfA*, *plcA, actA, Imo0673*, *bapL*, *recO, Imo2504,* and *luxS* play roles in the different stages of *L. monocytogenes* biofilm formation [35,46,69], though Price et al. [70] argued that the presence of LIPI-1 genes *hly* and *prfA* are required. However, biofilm formation is a complex and dynamic process that is contingent upon a number of factors, including the availability of nutrients in the environment, the origin and biodiversity of the strain, and quorum sensing (QS), which activate and regulate biofilm-associated genes and virulence factors [19]. Several studies have demonstrated that *L. monocytogenes* forms biofilms that exhibit significantly greater resistance to sanitizing and antibiotic compounds than free-floating cells [44,71]; therefore, it could represent a source of concurrent food contamination, thus increasing the risk to the consumer and impacting public health, in addition to the economic losses associated with voluntary recalls or damage to equipment within the food industry.

Moreover, in this study, *L. monocytogenes* strains isolated from fresh fruits and vegetables could have caused severe human infection; however, the severity of the clinical manifestation of *L. monocytogenes* is related to genetic diversity, immune system status, and host comorbidities. Indeed, in Mexico, listeriosis is not notified within the National Epidemiological Surveillance System, which has limited the characterization of the danger and risk it may pose for its population. Castañedas-Ruelas et al. [72] argued that the dearth of data concerning the significance of *L. monocytogenes* in Mexico underscores the necessity of sensitizing authorities to the risks associated with food and human exposure to *L. monocytogenes*, thereby facilitating an understanding of the clinical and epidemiological impacts of listeriosis in Mexico. Notably, it is essential to incorporate techniques with whole-genome sequencing (WGS) and multilocus sequence typing (MLST) that allow us to determine the biodiversity of *L. monocytogenes*, enabling the identification of clonal complexes (CCs), sublineages (SLs), and analysis of virulence genes of *L. monocytogenes* that contribute to the microbiological surveillance of Listeriosis, severity of the disease, sources, or the continuous improvement in control measures based on hazard characterization [34,46,73].

In addition to the continuous improvements in good agricultural practices, including the incorporation of agricultural or sanitary inputs such as phytogenic products, plant antimicrobial products can provide growth-promoting and pathogen-controlling effects, or substances generally recognized as safe (GRAS), such as citric acid, gallic acid, and lactic acid, can be incorporated, which show an antiplanktonic cell effect on *L. monocytogenes* or control of its biofilm, and can therefore be used among the disinfection methods implemented within the industry, with the intent of reducing antimicrobial resistance and its impacts on the environment and the consumer [46,74].

## 4. Materials and Methods

### 4.1. Sample Collection and Isolation of Listeria

Overall, 132 samples were collected, comprising 88 vegetables and 44 fruits, purchased from a local supermarket in Ocotlán, Jalisco, from May to August 2023. The raw vegetables and fresh fruits included Hass avocados, lettuce, parsley, cilantro, broccoli, and cucumber (twenty-two samples each). *L. monocytogenes* was isolated from foods according to the methods described in the Bacteriological Analytical Manual (individual subsample analysis, enrichment procedure, isolation procedure with Oxford agar (OXA; Becton Dickinson Bioxon, Le Pont de Claix, France) after 24–48 h incubation at 35 °C, with selection of up to 5 typical colonies for identification). The colonies were transferred into a tryptic soy broth (TSB; Becton Dickinson Bioxon, Le Pont de Claix, France) with 0.6% yeast extract (TSBYE), the incubated at 30 °C for 24–48 h and examined for morphological and biochemical characteristics using Gram staining, with hemolysis determined with 5% sheep blood agar, CAMP test, motility, catalase, and carbohydrate fermentation (mannitol, rhamnose, and xylose). Finally, the strains were confirmed via PCR using *hly* (listeriolysin O) and *prs* (putative phosphoribosyl pyrophosphate synthetase) [24,25,75]. Stocks were stored in TSB containing 30% glycerol at −80 °C.

### 4.2. Genomic Characterization: Genes Involved in Pathogenicity Islands, Biofilm Formation, and Antibiotic Resistance

*L. monocytogenes* strains were reactivated in TSBYE (Sigma-Aldrich, St. Louis, MO, USA) for 24 h at 30 °C. According to the manufacturer’s instructions, genomic DNA was extracted from *L. monocytogenes* using a Bacteria DNA Preparation Kit (Jena Bioscience, Jena, Germany). All *L. monocytogenes* strains were investigated for the detection of genes (*prfA*, *hly*, *plcA*, *plcB*, *mpl*, *actA*, *inlA*, *inlB*, *inlC*, *inlJ*, *llsA*, *llsG*, *llsH*, *llsX*, *llsB*, *llsY*, *llsD*, *llsP*, *licC*, *licB*, *licA*, and *glvA*) that harbored *L. monocytogenes* pathogenicity islands (LIPIs) via PCR using the protocol of Zhang et al. [21] (Table 4). Subsequently, *L. monocytogenes* phylogenetic groups (I.1 (172a-3a), I.2 (1/2c-3c), II.1 (4b-4d-4e), II.2 (1/2b-3b-7), and III (4a-4c)), and the genes associated with their antibiotic resistance (efflux pump Ide (*Ide*)*,* chloramphenicol acetyltransferase (*cat*)*,* macrolide-lincosamide-streptogramin B efflux pump (*msrA*), rRNA adenine-N-6-methyltransferase (*ermA*)*,* and ribosomal protection protein tetM (*tetM*)) were determined using the protocols of Doumith et al. [24] and Boháčová et al. [39]. After amplification, the products were electrophoresed on 1% (*w*/*v*) agarose gel (UltraPure agarose, Invitrogen, Carlsbad, CA, USA) using SYBR Green (Sigma-Aldrich, St. Louis, MO, USA) and visualized using transillumination under UV light (UVP, DigiDoc-It Darkroom, Upland, CA, USA).

### 4.3. Phenotypic Characterization for the Persistence of L. monocytogenes

#### 4.3.1. Disinfectant and Heavy Metal Sensitivity

Benzalkonium chloride (BC) (Sigma-Aldrich, St. Louis, MO, USA) was used to determine the sensitivity of *L. monocytogenes* strains to a quaternary ammonium compound (QAC) using the protocol of Gray et al. [30] with modifications. *L. monocytogenes* strains were grown overnight in Mueller–Hinton broth (MHB; Becton Dickinson Bioxon, Le Pont de Claix, France) at 30 °C and subsequently diluted to ~10^8^ CFU/mL. A BC stock concentration of 100 µL was added to the microtiter plates (Corning^®^ 96-Well Assay Microplate, Lowell, MA, USA) with concentrations of 100, 50, 25, 12.5, 6.2, 3.1, 1.5, and 0.7 µg/mL. The microtiter plates were then incubated at 30 °C/24 h, and growth was monitored by measuring the OD_560_ using a Multiskan FC (Thermo Fisher Scientific, Inc., Madison, WI, USA) in order to determine the minimum inhibitory concentration (MIC). Each strain was tested in triplicate, with the positive (100 µL of MHB + 100 µL of *L. monocytogenes* ATCC 19111 (~10^8^ CFU/mL)) and negative control wells containing only 200 µL of MHB. Cadmium chloride (CdCl_2_; Sigma-Aldrich, St. Louis, MO, USA) was used to determine the resistance of *L. monocytogenes* to the heavy metal cadmium. Mueller–Hinton agar (MHA; Becton Dickinson Bioxon, Le Pont de Claix, France) was supplemented with different concentrations of CdCl_2_ (400, 200, 100, 70, 50, 25, and 12.5 µg/mL); each *L. monocytogenes* isolate was adjusted to ~10^8^ CFU/mL and inoculated onto the CdCl_2_ plates, which were then incubated at 37 °C/24 h in triplicate. Resistance to cadmium was interpreted as ≥70 µg/mL [49,50].

#### 4.3.2. Phenotypic Antibiotic Sensitivity and Resistance Analysis

The antibiotic resistance and susceptibility of the *L. monocytogenes* strains were determined using the agar diffusion method, following guidelines from the Clinical and Laboratory Standards Institute (CLSI) [76]. Bacterial suspensions, adjusted to 0.5 McFarland, were inoculated onto MHA, where antibiotics were incorporated, and the specimens were incubated at 35 °C/24 h. Thereafter, among the eleven classes of antimicrobials, the following thirteen antibiotics were selected for testing: phenicols (chloramphenicol (CL, 30 µg)); cephalosporines (1st generation) (cephalothin (CF, 30 µg)); lincosamides (clindamycin (CLM, 30 µg)); sulfonamides (trimethoprim–sulfamethoxazole (SXT, 2.5/23.75 μg)); cyclic peptides (tetracycline (TE, 30 µg)); macrolides (erythromycin (E, 15 µg)); aminoglycosides (gentamicin (GE, 10 µg)); fluoroquinolones (ciprofloxacin (CPF, 5 μg)); cephalosporines (3rd generation) (cefotaxime (CFX, 30 µg)); glycopeptides (vancomycin (VA, 30 µg)); and β-lactams (penicillin (P, 10 U), ampicillin (AM, 10 µg), and dicloxacillin (DC, 1 µg)) (BBL^TM^ Sensi-Disc^TM^). The inhibition zones were interpreted as resistance (R), intermediate resistance (I), and susceptible (S), according to CLSI [76]. *L. monocytogenes* ATCC 19111 was used as the positive control. The multiple antibiotic resistance (MAR) index of each *L. monocytogenes* isolate was determined using the methods described by Krumperman [41] and Blasco et al. [77]; the MAR index is defined as *a*/*b*, where *a* is the number of antibiotics the isolate was resistant to, and *b* represents the total number of antibiotics to which the isolate was exposed.

#### 4.3.3. Biofilm Formation Assay

Each strain’s ability to form a biofilm was evaluated in a polystyrene microtiter plate (Corning^®^ 96-Well Assay Microplate, Lowell, MA, USA) using crystal violet (CV) staining, following the protocol described by Avila-Novoa et al. [78]. For each strain, 230 μL of TSB and 20 μL bacterial suspension (~10^8^ CFU/mL) were added to a polystyrene microtiter plate and incubated at 30 °C for 240 h. The planktonic bacteria were removed using 200 μL of phosphate-buffered saline (PBS; 7 mM Na_2_HPO_4_, 3 mM NaH_2_PO_4_, and 130 mM NaCl, pH 7.4). The biofilm was fixed with 200 μL of methanol for 10 min, dried at 55 °C for 15 min, and stained with 200 µL of 0.1% crystal violet for 45 min. Excess stain was rinsed off with PBS and resolubilized with 200 μL of 95% ethanol. Absorbance was measured at 570 nm (OD_570_) using the Multiskan FC. The assay was performed in triplicate, including positive (230 μL of TSB and 20 μL of *L. monocytogenes* ATCC 19111 (~10^8^ CFU/mL)) and negative control wells, which contained TSB only. The cut-off O.D. (O.D.c) value was determined using the protocol described by Stepanović et al. [79], defined as three standard deviations above the mean O.D. of the negative control. Based on the O.D. values of the bacterial films, strains were classified into the following categories: nonbiofilm producers (O.D. < O.D.c), weak biofilm producers ((O.D. c < O.D.c < (2 × O.D.c)), moderate biofilm producers ((2 × O.D.c) < O.D. < (4 × O.D.c)), and strong biofilm producers ((4 × O.D.c) < O.D.).

## 5. Conclusions

In the present study, we obtained data on virulence-associated genes (LIPI-1, LIPI-2, LIPI-3, and LIPI-4) and resistance mechanisms to antibiotics used in clinical or veterinary medicine, as well as the environmental impacts of fertilizer residues on the antimicrobial resistance of *L. monocytogenes* isolated from fruits and vegetables. With this study, we aimed to raise awareness of the continuous improvement needed regarding treatments and sanitary prerequisites in areas such as agricultural practices, food farming practices, and standard operating procedures for sanitation. Future research should consider vali-dating and rotating disinfectants to reduce the risk of *L. monocytogenes* niche establishments in the environment and disinfectant tolerance that promotes the survival of *L. monocytogenes* biofilms. Additionally, a genomic analysis should be conducted of *L. monocytogenes* considering various sources of contamination (humans, animals, food, and the environment) within the traceability (production, transformation, and distribution of food) of fruits and vegetables to characterize the biological hazard, associate the source of contamination, and establish an efficient control measure to reduce the risk to the consumer.

## Figures and Tables

**Table 1:** Genetic analysis and antibiotic resistance of *L. monocytogenes* isolates from fruits and vegetables.

Strain No.	Sample	Genetic Determinants of Virulence	Phylogenetic Group	Serotype	CdCl_2_	CPF	Antimicrobial Resistance Genes	Antibiotic Resistance Pattern	MAR Index
Lm-11	Cilantro	LIPI-1 + LIPI-2	II.2	1/2b	R	S	*---*	PE-CF-AM-CFX-DC	0.38
Lm-14	Broccoli	LIPI-1 + LIPI-2	II.2	1/2b	R	I	*Ide*	PE-CF-AM-CFX-DC	0.38
Lm-13	Lettuce	LIPI-1 + LIPI-2	II.2	1/2b	R	S	*msrA + tetM*	PE-CF-AM-CFX-DC-TE	0.46
Lm-17	Lettuce	LIPI-1 + LIPI-2	II.2	1/2b	R	I	*Ide*	PE-CF-AM-CFX-DC	0.38
Lm-18	Cilantro	LIPI-1 + LIPI-2	II.2	1/2b	R	I	*Ide*	PE-CF-AM-CFX-DC-CLM	0.46
Lm-42	Hass avocados	LIPI-1 + LIPI-2	II.2	1/2b	S	S	*tetM*	PE-CF-AM-CFX-DC-CLM-TE	0.53
Lm-43	Hass avocados	LIPI-1 + LIPI-2	II.2	1/2b	S	S	*---*	PE-CF-AM-CFX-DC-TE	0.46
Lm-68	Hass avocados	LIPI-1 + LIPI-2	II.2	1/2b	S	I	*Ide + msrA*	PE-CF-AM-CFX-DC	0.38
Lm-136	Hass avocados	LIPI-1 + LIPI-2	II.2	1/2b	S	I	*Ide*	PE-CF-AM-CFX-DC-CLM	0.46
Lm-133	Hass avocados	LIPI-1 + LIPI-2	II.2	1/2b	S	S	*---*	PE-CF-AM-CFX-DC-CLM	0.46
Lm-147	Hass avocados	LIPI-1 + LIPI-2	II.2	1/2b	S	R	*Ide + tetM*	PE-CF-AM-CFX-DC-CPF-CLM-TE	0.61
Lm-138	Hass avocados	LIPI-1 + LIPI-2	II.2	1/2b	R	I	*Ide*	PE-CF-AM-CFX-DC-CLM	0.46
Lm-19	Lettuce	LIPI-1 + LIPI-2 + LIPI-3	I.1	1/2a	S	S	*tetM*	PE-CF-AM-CFX-DC-TE	0.46
Lm-24	Lettuce	LIPI-1 + LIPI-2 + LIPI-3	I.1	1/2a	R	S	*---*	PE-CF-AM-CFX-DC-CLM	0.46
Lm-15	Broccoli	LIPI-1 + LIPI-2 + LIPI-3	I.1	1/2a	R	S	*msrA*	PE-CF-AM-CFX-DC-CLM	0.46
Lm-27	Lettuce	LIPI-1 + LIPI-2 + LIPI-3 + LIPI-4	I.1	1/2a	R	R	*Ide + tetM*	PE-CF-AM-CFX-DC-CPF-CLM-TE	0.61
Lm-41	Broccoli	LIPI-1 + LIPI-2 + LIPI-3 + LIPI-4	I.1	1/2a	R	I	*Ide*	AM-CFX-DC	0.23

LIPI-1, isolates harboring virulence genes *prfA, hly, plcA, plcB, mpl,* and *actA*; LIPI-2, isolates harboring virulence genes *inlA, inlB, inlC,* and *inlJ;* LIPI-3, isolates harboring virulence genes *llsA, llsG, llsH, llsX, llsB, llsY, llsD,* and *llsP;* LIPI-4, isolates harboring virulence genes *licC, licB, licA*, and *glva*; I, intermediate resistance to ciprofloxacin; R, resistance to ciprofloxacin or CdCl_2_; S, susceptible to ciprofloxacin or CdCl_2_; CPF, ciprofloxacin; AM, ampicillin; CLM, clindamycin; CF, cephalothin; CFX, cefotaxime; CL, chloramphenicol; GE, gentamicin; E, erythromycin; TE, tetracycline; PE, penicillin; DC, dicloxacillin. MAR, multiple antibiotic resistance.

**Table 2:** Antimicrobial susceptibility test for *L. monocytogenes*.

Antimicrobial Class According to the WHO		Antibiotic ^1^	No. (%) of *L. monocytogenes* Strains		
			Resistant	Susceptible	Intermediate
Highly important	Phenicols	CL		100	
	Cephalosporines (1st generation)	CF	94.1		5.8
	Lincosamides	CLM	52.9	5.8	41.1
	Sulfonamides	SXT		100	
	Cyclic peptides	TE	29.4	70.5	
Critically important	Macrolides	E		94.1	5.8
	Aminoglycosides	GE		94.1	5.8
	Fluoroquinolones	CPF	11.7	47	41.1
	Cephalosporines (3rd generation)	CFX	100		
	Glycopeptides	VA		100	
	β-Lactams	DC	100		
		AM	100		
		PE	94.1	5.8	

^1^ AM, ampicillin; CLM, clindamycin; CF, cephalothin; CFX, cefotaxime; CPF, ciprofloxacin; CL, chloramphenicol; GE, gentamicin; E, erythromycin; TE, tetracycline; VA, vancomycin; SXT, trimethoprim–sulfamethoxazole; PE, penicillin; DC, dicloxacillin.

**Table 3:** Minimum inhibitory concentration values of BC and CdCl_2_ in *L. monocytogenes* strains in relation to biofilm formation.

Strain No.	Phylogenetic Group	Serotype	MIC (µg/mL)	BC	CdCl_2_	Biofilm Formation (Microtiter Plate Assays)
Lm-11	II.2	1/2b	6.2	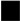	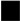	Moderate biofilm
Lm-14	II.2	1/2b	6.2	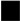	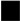	Moderate biofilm
Lm-13	II.2	1/2b	6.2	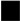	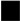	Moderate biofilm
Lm-17	II.2	1/2b	6.2	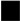	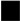	Moderate biofilm
Lm-18	II.2	1/2b	3.1	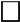	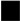	Moderate biofilm
Lm-27	I.1	1/2a	3.1	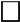	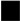	Moderate biofilm
Lm-42	II.2	1/2b	3.1	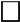	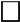	Moderate biofilm
Lm-43	II.2	1/2b	3.1	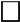	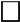	Moderate biofilm
Lm-68	II.2	1/2b	3.1	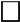	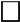	Moderate biofilm
Lm-133	II.2	1/2b	3.1	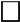	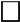	Moderate biofilm
Lm-136	II.2	1/2b	3.1	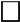	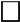	Moderate biofilm
Lm-138	II.2	1/2b	1.5	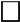	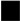	Moderate biofilm
Lm-41	I.1	1/2a	0.7	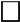	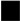	Moderate biofilm
Lm-147	II.2	1/2b	0.7	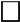	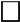	Moderate biofilm
Lm-19	I.1	1/2a	0.7	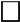	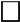	Moderate biofilm
Lm-24	I.1	1/2a	1.5	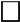	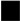	Moderate biofilm
Lm-15	I.1	1/2a	1.5	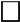	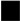	Moderate biofilm

Black box indicate resistance to BC (CMI ≥ 6 µg/mL) or CdCl_2_ (CMI ≥ 70 µg/mL).

**Table 4:** Primers for the amplification of *L. monocytogenes* virulence-associated genes.

Gene	Primer Sequences (5′-3′)	Protein Coded by Target Gene (Gene)	Biological Function	References
LIPI-1				
*prfA*	F: 5′-AACGGGATAAAACCAAAACCA-3′ R: 5′-TGCGATGCCACTTGAATATC-3′	Transcriptional regulator A (prfA)	Controls and regulates the expression levels of *L. monocytogenes* virulence factors.	[21,29]
*hly*	F: 5′-GTTAATGAACCTACAAGACCTTCC-3′ R: 5′-ACCGTTCTCCACCATTCCCA-3′	Listeriolysin O (hly)	Phagosome lysis.	[21,26,27,28]
*plcA*	F: 5′-TCCCATTAGGTGGAAAAGCA-3′ R: 5′-CGGGGAAGTCCATGATTAGA-3′	Phosphatidyl inositol phospholipase C (plcA)	Phagosome lysis.	[21,26,27,28]
*plcB*	F: 5′-CAGCTCCGCATGATATTGAC-3′ R: 5′-CTGCCAAAGTTTGCTGTGAA-3′	Phosphatidyl choline phospholipase C (plcB)	Phagosome lysis.	[21,26,27,28]
*mpl*	F: 5′-AAAGGTGGAGAAATTGATTCG-3′ R: 5′-AGTGATCGTATTGTAGGCTGCTT-3′	Metalloprotease (mpl)	Processes the PC-PLC precursor to its mature form.	[21,26,27,28]
*actA*	F: 5′-AAACAGAAGAGCAGCCAAGC-3′ R: 5′-TTCACTTCGGGATTTTCGTC-3′	Protein for actin nucleation (actA)	Facilitates the motility of the bacterial cell to the host cell’s cytoplasm.	[21,26,27,28]
LIPI-2				
*inlA*	F: 5′-ACGAGTAACGGGACAAATGC-3′ R: 5′-CCCGACAGTGGTGCTAGATT-3′	Internalin A (inlA)	Adhesion and invasion of *L. monocytogenes* cells to the host cell and dissemination.	[5,7,21,22,29,30,31]
*inlB*	F: 5′-CATGGGAGAGTAACCCAACC-3′ R: 5′-GCGGTAACCCCTTTGTCATA-3′	Internalin B (inlB)	Adhesion and invasion of *L. monocytogenes cells* to the host cell, and dissemination.	[5,7,21,22,29,30,31]
*inlC*	F: 5′-AATTCCCACAGGACACAACC-3′ R: 5′-CGGGAATGCAATTTTTCACTA-3′	Internalin C (inlC)	Contribute postintestinal stages of infection.	[6,21,22,32]
*inlJ*	F: 5′-TGTAACCCCGCTTACACACAGTT-3′ R: 5′-AGCGGCTTGGCAGTCTAATA-3′	Internalin J (inlJ)	Involved in passage through the intestinal barrier, as well as in subsequent stages of infection.	[6,21,22,32]
LIPI-3				
*llsA*	F: 5′-ATGAATATTAAATCACAATCATCA-3′ R: 5′-TTACATTTTGGTTGCAGCAG-3′	Operon coding LLS (contributes to the expression of listeriolysin S (LLS))	Bacteriocin, with hemolytic and cytotoxic factors that alter the host intestinal microbiota and promote the survival of *L. monocytogenes* in polymorphonucleocytes.	[21,22,30,33]
*llsG*	F: 5′-GAGACTGGGCTTACTTGC-3′ R: 5′-TACCTCCTGTTCACTGCTTG-3′			
*llsH*	F: 5′-ATGATGTTCGCTATGGTT-3′ R: 5′-ACATTCCTACTGGCATCA-3′			
*llsX*	F: 5′-TTATTGCATCAATTGTTCTAGGG-3′ R: 5′-CCCCTATAAACATCATGCTAGTG-3′			
*IIsB*	F: 5′-TTACAATCAACCACCAGG-3′ R: 5′-AGTGAACCGAATGACAGA-3′			
*IIsY*	F: 5′-ATTAGAATAGGAACGCAGAC-3′ R: 5′-TCATAGCACCCAGTTTCG-3′			
*IIsD*	F: 5′-TATGGTGGTATGGAGGGT-3′ R: 5′-ATCACCCTGCTTATTTCA-3′			
*IIsP*	F: 5′-TTTCCAGGTATGCTTCTT-3′ R: 5′-CAATTACGGTGGTTCTCA-3′			
LIPI-4				
*licC*	F: 5′-GGGATTCCGAAACTACCT-3′ R: 5′-CGAGTGCTCCTGTAACCC-3′	Cellobiose family phosphotransferase system (PTS)	Involved in infection of the host’s neuronal and placental tissues.	[21,34,35]
*licB*	F: 5′-ATTGCGGCATCTGAGAAA-3′ R: 5′-CAGCGATTAGAATTGGTACTGC-3′			
*licA*	F: 5′-GCCTCTTCCTCGTTTCTA-3′ R: 5′-GACTTAACTAAATCGCAGTA-3′			
*glvA*	F: 5′-TTACTATTGCTGGCGGAGGA-3′ R: 5′-TGCTCACGACCATCCATT-3′			

## Data Availability

The data used to support the findings of this study are available from the corresponding author upon request.
